# Facility level factors that determine consistent delivery of essential newborn care at health centers in Ethiopia

**DOI:** 10.1186/s12884-021-04358-4

**Published:** 2022-01-16

**Authors:** Binyam Fekadu, Ismael Ali, Zergu Tafesse, Hailemariam Segni

**Affiliations:** JSI Research and Training Institute, Inc, USAID Transform: Primary Health Care, Addis Ababa, Ethiopia

**Keywords:** Essential newborn care, ENC, Primary health care, Providers’ attitude

## Abstract

**Background:**

Essential newborn care (ENC) is a package of interventions which should be provided for every newborn baby regardless of body size or place of delivery immediately after birth and should be continued for at least the seven days that follows. Even though Ethiopia has endorsed the implementation of ENC, as other many counties, it has been challenged. This study was conducted to measure the level of essential newborn care practice and identify health facility level attributes for consistent delivery of ENC services by health care providers.

**Methods:**

This study employed a retrospective cross-sectional study design in 425 facilities. Descriptive statistics were formulated and presented in tables. Binary logistic regression was employed to assess the statistical association between the outcome variable and the independent variables. All variables with *p* < 0.2 in the bivariate analysis were identified as candidate variables. Then, multiple logistic regression analysis was performed using candidate variables to determine statistically significant predictors of the consistent delivery of ENC by adjusting for possible confounders.

**Results:**

A total of 273, (64.2%), of facilities demonstrated consistent delivery of ENC. Five factors—availability of essential obstetrics drugs in delivery rooms, high community score card (CSC) performances, availability of maternity waiting homes, consistent partograph use, and availability of women-friendly delivery services were included in the model. The strongest predictor of consistent delivery of essential newborn care (CD-ENC) was consistent partograph use, recording an odds ratio of 2.66 (AOR = 2.66, 95%CI: 1.71, 4.13). Similarly, providing women-friendly services was strongly associated with increased likelihood of exhibiting CD-ENC. Furthermore, facilities with essential obstetric drugs had 1.88 (AOR = 1.88, 95%CI: 1.15, 3.08) times higher odds of exhibiting consistent delivery of ENC.

**Conclusion:**

The delivery of essential newborn care depends on both health provider and facility manager actions and availability of platforms to streamline relationships between the clients and health facility management.

## Background

World leaders have renewed their commitment to further reduce neonatal mortality as part of the sustainable development goals (SDGs). A new goal (SDG Target 3.2) has been set to make sure that every country works towards reducing neonatal mortality to at least 12 per 1,000 live births by 2030 [1]. Studies show that in developing countries, the causes of death and the likelihood of exposure to those causes can dramatically be minimized and avoided with simple, low-cost and time-saving measures [2, 3]. Nearly all neonatal deaths in developing countries are attributed to three main causes: complications of preterm birth, asphyxia, and infection [4]. Improving the quality of care for newborns is thus important to ensuring their survival, growth, and development [5].

Essential newborn care (ENC) is a package of interventions which should be provided for every newborn baby regardless of body size or place of delivery immediately after birth and should be continued for at least the seven days that follow [6]. It is comprised of a package of simple, evidence-based interventions proven to be effective in preventing or treating the most common causes of newborn morbidity and mortality [7]. Ethiopia has endorsed the implementation of ENC as one of the critical elements for newborn health. The interventions include appropriate cord care, provision of vitamin K and TTC eye ointment, optimal thermal care and early breastfeeding within one hour of birth as well as counseling on feeding practices. Optimal thermal care is defined as a baby wrapped within 10 min of birth, receiving a first bath after six or more hours and the use of warm water to bathe the baby [1].

Although a minimum package of proven interventions to reduce newborn mortality have been adopted, countries are still challenged by multiple system related problems [8]. Provision of good quality care depends on service availability, work environment and overall system support [9]. As underlined by various studies, consistent delivery of the ENC is important. However, it is affected by workload, training status of health workers, and the availability of materials (e.g. guidelines, drugs, etc.) [5]. While working on expanding the availability of the packages, a focus on improving their functionality and utilization is imperative [10]. This requires regular measurement to further improve the quality of the implementation of the service packages [11]. Improvements in most of the key practices do not require large investments in equipment or supplies [8].

Reviewing the current practices with regards to newborn care, suboptimal newborn care practices continue to occur which denotes limited or no improvements in the reduction of neonatal mortality rates [1]. The situation in Ethiopia is no different. Neonatal mortality declined from 39 deaths per 1,000 live births in 2005 to 29 deaths per 1,000 live births in 2016, a reduction of 10% over 11 years [12]. The rate then increased to 30/1,000 live births in 2019 [13]. Solving this problem requires an understanding of the national guidance on newborn care and further assessing facility readiness, health worker competencies, health worker-patient interactions, and work environments [14].

USAID Transform: Primary Health Care project has developed a package of interventions to improve the quality of newborn care services at the health center level. The interventions include provision of trainings to care providers, strengthening the availability of newborn corners, on-site mentorship and supervision support, and strengthening the referral and feedback system between primary hospitals and health centers. Despite the package of interventions, the practice of ENC varies between facilities. This study was conducted to measure the level of essential newborn care practice and identify associations of health facility level infrastructure, health workforce, readiness, interactions and experiences of care attributes for consistent delivery of ENC services by health care providers.

## Methods

### Study settings

The study area covers 1,880 health centers in the 396 intervention districts of the USAID Transform: Primary Health Care project [15].

### Study design and instruments

The study employed a retrospective cross-sectional study design and used program monitoring data collected from October to December 2019. During this period, a total of 425 visits were made to health centers selected using simple random sampling method. The study analyzed the 425 supportive supervision visits data made to the health centers during the three-month period.

### Data collection

Three types of data collection procedure are used during an on-site technical assistance visit. The first inquiries about health worker knowledge on the practices of essential newborn care services using a close-ended individual interview questions, the second is a simple observation of the availability of equipment and other relevant materials for care in the facility physical setting and the third involves a retrospective review of patient medical records by extracting last three months recorded data. The data collection is supported by an online electronic system [15]. For this study, data collected using the three procedures were used for analysis.

### Variables of interest

The dependent variable of the study is consistent delivery of ENC by health care providers. Using data pulled from the medical records, the facilities practicing all the ENC components to all cases were labeled as “yes” and those that do not practice all, labeled as a “no”. The independent variable for this study includes other facility and input level variables (Table 1). They were included in the study to identify facility level attributes for observed differences between facilities in the consistent delivery of ENC. The variables based on findings from other studies [1, 5, 16] and program implementation experience are described in the table below (Table 1).

**Table 1:** Variables included in the analysis

**Category**	**Components**
**Infrastructure related factors**	•Distance between facility and woreda capital (KM) •Access to roads
**Health workforce related factors**	•Availability of trained providers •Availability of technical staff as per the standard •Consistent partograph use
**Facility readiness**	•Availability of water •Availability of electricity •Presence of all the required laboratory investigations for antennal care •Availability of maternity waiting homes •Basic emergency obstetrics and newborn care (BEmONC) signal functions •Separate delivery rooms •Availability of essential obstetric drugs in delivery rooms (vitamin K, Tetracycline eye ointment and other obstetric drugs) •Availability of newborn corners •Established case review/audit system for maternal and newborn deaths
**Interactions and experiences of care**	•High caseload facility •Community scorecard performance •Availability of women-friendly delivery services

### Operational definition of terms

#### Consistent delivery of essential newborn care (CD-ENC)

A central goal of the project and the objective for its support to health workers and the health system. Essential newborn care is the care provided to the neonate after birth within the delivery room by skilled personnel which includes vitamin k provision, eye care, cord care, initiating exclusive breastfeeding, and thermal care. And, it is considered as consistent delivery when all the care packages are delivered to the newborn. The data collectors randomly pick five cards from the months prior to the data collection date and check for what is recorded on the patient card. After review of each card, the data collectors then mark if ENC is practiced or not. The case is marked as ‘yes’ when all the five components of the package, (vitamin K, TTC eye ointment, chlorohexidine for cord care, early initiation of breastfeeding and thermal care) are recorded as having been provided.

#### Availability of trained providers

The facility having two or more providers trained on either BEmONC or ENC within the last three years.

#### Availability of technical staff as per the standard

The facility having 25 or more technical staff that provide services. The technical staff are health professionals that are assessing and managing medical treatments, providing ongoing care, and providing services to help with diagnosis and treatment.

#### High caseload facility

The facility offering delivery services for more than 500 births per year.

#### Community scorecard (CSC) performance (Favorable user experiences)

A continuous variable—the community analyzes facility services and score them based on their personal perceptions of the services. As per the Ethiopian standard operating procedure, selected community representatives rate each facility’s performance based on the selected six indicators (1) compassionate, respectful and caring health workforce; (2) availability of services, biomedical equipment and pharmaceutical supplies; (3) patient waiting time; (4) health facility infrastructure; (5) ambulance service and management; and (6) clean and safe health facility (CASH). A composite ratio scale of the six indicators was used for analysis.

#### Availability of newborn corners

A facility is labeled as having newborn corners when it has all the required equipment and materials in the labor and delivery rooms. The equipment and materials are: Ambu bag, suction machine, radiant warmer/heat source, oxygen source, fixed length board, resuscitator, weight scale, thermometer, stethoscope, mucus extractor, towel, cord care equipment, tube for feeding, sterile gloves, TTC eye ointment, vitamin K, CHX, syringe, feeding cup, ampicillin, and gentamicin.

#### Consistent use of partograph

A facility which exhibits correct use of partographs in the reviewed patient cards.

#### Have all the required laboratory investigations for ANC

The facility having tests for Rh blood group, hemoglobin, venereal disease research laboratory (VDRL)/RPR (rapid plasma reagin), urinalysis, provider initiated testing and counseling (PITC) for HIV, and hepatitis B surface antigen (HBsAg).

#### BEmONC signal functions

Basic emergency obstetric and newborn care (BEmONC) is a primary health care level initiative promoted in low and middle-income countries to reduce maternal and newborn mortality. A set of seven key obstetric services, or “signal functions,” has been identified as critical to BEmONC. BEmONC is recorded as “Yes” if all the seven signal functions, 1) Administration of parenteral antibiotics, 2) Administration of parenteral anticonvulsants, 3) Administration of parenteral uterotonics, 4) Removal of retained products (manual vacuum aspiration), 5) Assisted vaginal delivery, 6) Manual removal of the placenta, and 7) Resuscitation of the newborn are provided are available and recorded as “No” If any of the signal functions are missing.

### Data analysis

Data were managed using a web-based system, DHIS2 [17], and then exported to SPSS version 25 for statistical analysis. Descriptive statistics were formulated and presented in tables. Binary logistic regression was employed to assess the statistical association between the outcome variable and the independent variables. First, the assumptions including multicollinearity among the independent variables and linearity of independent variables for binary logistic regression model were checked and then bivariate analysis was used to identify candidate variables for multiple logistic regression analysis. All variables with *p* < 0.2 in the bivariate analysis were identified as candidate variables. Then, multiple logistic regression analysis was performed using candidate variables to determine statistically significant predictors of the consistent delivery of ENC by adjusting for possible confounders. In addition, a variable that was significant from a program implementation point of view was included in the final model even if the bivariate inclusion criteria were not met. Finally, variables with a p value less than 0.05 from the logistic regression were declared as statistically significant. Adjusted odds ratio with 95% CI was estimated to identify predictors for consistent delivery of ENC. Multicollinearity between the study variables was diagnosed using a variance inflation factor (VIF), an eigenvalue, and a condition index. Large, greater than 5, VIF values indicate a high degree of collinearity or multicollinearity among the independent variables [18]. Linearity of the continuous variables, distance between facility and woreda capital (KM) and community score card, with respect to the logit of the dependent variables were assessed via the Box-Tidwell (1962) procedure. The goodness-of-fit of the model was also checked using the Hosmer–Lemeshow test.

### Ethical considerations

The study used project data that has been collected as part of follow-up monitoring visits to health centers. The results of the study did not distinguish the name of the district and other specific site identifiers. Therefore, JSI research and Training Institute, Inc’s Institutional Review Board (IRB) has determined that this activity is exempt from human subjects’ oversight (IRB #20-17E). As part of the activity facility entry and document review permissions were sought from the health center management and staff.

## Results

### Description of facilities

A total of 425 health centers were included in the study. Most of the facilities—186 (43.8%)—were in the Oromia region. Most facilities were located more than 5 km away from district capitals, 331 (77.9%), with an average distance of 21.7 ± 24.2 standard deviation (SD) kilometers from a facility to the district capital. Four hundred and one, (94.4%) of the facilities have access to roads and 121, (28.5%) of facilities were staffed as per the standard (Table 2). The average catchment population of the study facilities was 25,249.8 ± 12,467.1 SD.

**Table 2:** Characteristics of facilities in the study (*n* = 425)

Characteristics	Number (percent)
Facility distribution by region	
Amhara	89 (20.9)
Oromia	186 (43.8)
SNNP	87 (20.5)
Tigray	63 (14.8)
Facility location	
> 5 km away from the district capital	331 (77.9)
within 5 km of the district capital	94 (22.1)
Access to roads	401 (94.4%)
Availability of technical staff as per the standard	121 (28.5)

### Description of facilities that are consistently applying essential newborn care

Of the total 425 facilities, 273 (64.2%), consistently delivered ENC. The mean distance between facilities consistently delivering ENC from the woreda capital was 21.2 ± 23.3 SD. A significant proportion of them, 254 (94.1%), had access to an all-weather road. With regards to human resources among facilities with consistent delivery of ENC, 161 (59.0%), had two or more providers trained on either BEmONC or ENC. Facilities that have 25 or more technical staff who provide services constituted 28.6% of the facilities practicing consistent delivery of ENC. Most of the facilities that were practicing consistent delivery of ENC were also using partographs consistently (Table 3).

**Table 3:** Bivariate logistic regression analysis

Predictor		Consistent delivery of essential newborn care		Total (%) or Mean (SD)	COR	95% CI	P-Value
		No	Yes				
		Mean (SD) or No (%)	Mean (SD) or Yes (%)				
Distance between facility and woreda capital (KM)		22.4 (25.8)	21.2 (23.3)	21.7(24.2)	1.00	0.99—1.01	0.614
Access to roads (1)	No	8 (5.3)	16 (5.9)	24 (5.7)	0.89	0.37—2.14	0.798
	Yes	144 (94.7)	257 (94.1)	401 (94.4)			
	Total	152 (100)	273 (100)	425 (100)			
Availability of trained providers (1)	No	71 (46.7)	112 (41.0)	183 (43.1)	1.26	0.84—1.88	0.257^a^
	Yes	81 (53.3)	161 (59.0)	242 (64.2)			
	Total	152 (100)	273 (100)	425 (100)			
Availability of technical staff as per the standard (1)	No	109 (71.7)	195 (71.4)	304 (71.5)	1.01	0.65—1.57	0.951
	Yes	43 (28.3)	78 (28.6)	121 (28.5)			
	Total	152 (100)	273 (100)	425 (100)			
Consistent partograph use (1)	No	88 (57.9)	83 (30.4)	171 (40.2)	3.15	2.08—4.75	0.000^a^
	Yes	64 (42.1)	190 (69.6)	254 (59.8)			
	Total	152(100)	273(100)	425(100)			
Water (1)	No	64 (42.1)	109(39.9)	173(40.7)	1.09	0.73—1.64	0.661
	Yes	88 (57.8)	164(57.8)	252(59.3)			
	Total	152(100)	273(100)	425 (100)			
Electricity (1)	No	43 (28.2)	68 (24.9)	111 (26.1)	1.19	0.76—1.86	0.447
	Yes	109 (71.7)	205 (71.7)	314 (73.9)			
	Total	152(100)	273(100)	425(100)			
Availability of all the required laboratory investigations for ANC available (1)	No	65 (42.8)	88 (32.2)	153 (36)	1.57	1.04—2.37	0.031^a^
	Yes	87 (57.2)	185 (67.8)	272 (64)			
	Total	152(100)	273(100)	425(100)			
Availability of maternity waiting homes (1)	No	42 (27.6)	50 (18.3)	92 (21.6)	1.70	1.06—2.72	0.026^a^
	Yes	110 (72.3)	223 (81.6)	333 (78.4)			
	Total	152(100)	273(100)	425(100)			
Administration of BEmONC signal functions (1)	No	51 (33.5)	53 (19.4)	104 (24.5)	2.10	1.34—3.29	0.001^a^
	Yes	101 (66.4)	220 (80.5)	321 (75.5)			
	Total	152(100)	273(100)	425(100)			
Separate delivery rooms (1)	No	15 (9.9)	15 (5.5)	30 (7.1)	1.88	0.89—3.97	0.096^a^
	Yes	137 (90.1)	258 (94.5)	395 (92.9)			
	Total	152(100)	273(100)	425(100)			
Availability of essential obstetric drugs in delivery rooms (1)	No	79 (51.9)	80 (29.3)	159 (37.3)	2.61	1.73—3.94	0.000^a^
	Yes	73 (48)	193 (70.6)	266 (62.6)			
	Total	152(100)	273(100)	425(100)			
Availability of newborn corners (1)	No	56 (36.8)	71 (26.0)	127 (29.9)	1.66	1.08—2.54	0.020^a^
	Yes	96 (63.2)	202 (74.0)	298 (70.1)			
	Total	152(100)	273(100)	425(100)			
Established case review/audit system for maternal and newborn deaths (1)	No	84 (55.3)	120 (44.0)	204 (48.0)	1.57	1.06—2.35	0.026^a^
	Yes	68 (44.7)	153 (56.0)	221 (52.0)			
	Total	152 (100)	273 (100)	425 (100)			
High caseload facility (1)	No	108 (71.1)	199 (72.9)	307 (72.2)	0.91	0.59—1.42	0.685
	Yes	44 (28.9)	74 (27.1)	118 (27.8)			
	Total	152 (100)	273 (100)	425 (100)			
CSC performance		53.8 (30.1)	46.1 (32.9)	48.8(32.1)	0.99	0.99—1.00	0.022^a^
Provision of women-friendly delivery services (1)	No	31 (20.4)	21 (7.7)	52 (12.2)	3.07	1.70—5.57	0.000^a^
	Yes	121 (79.6)	252 (92.3)	373 (87.8)			
	Total	152 (100)	273 (100)	425 (100)			

(1) Nos were coded "0" and yeses as "1"; first category was used as reference

^a^Candidate variable that were considered for entry into final model with *p* < 0.2

*ANC* Antenatal care, *BEmONC* Basic Emergency Obstetrics and Neonatal Care, *CSC* Community Score Card, *KM* Kilometers, *SD* Standard Deviation

As per this study’s findings, 164 (57.8%) and 205 (71.7%) had access to potable water and source of electricity, respectively. Health facilities’ service availability during ANC is designed to strengthening the relationship between clients and the service providers. In this regard, 185 (67.8%), of facilities reported availability of essential laboratory investigations for ANC and 223, (81.6%) reported having maternity waiting homes within the health centers. In addition, the readiness of the health centers for quality delivery care was also checked. The seven BEmONC signal functions were available in 220, (80.5%) of the health centers that were practicing ENC consistently. In addition, 258, (94.5%) and 202, (74%) of health centers had separate delivery rooms and newborn corners at the time of the visits, respectively. Moreover, for creating easy access to health workers, 193, (70.6%) of health centers availed essential obstetric drugs in the delivery rooms, and 153, (56%) of health centers had established a committee to audit maternal and perinatal deaths (Table 3).

The friendliness of care is also an important factor for ensuring ENC. Seventy-four, (27.1%) had high caseloads, and 252, (92.3%) of facilities had women-friendly delivery services. The average CSC performance of facilities practicing consistent delivery of ENC was 53.6 ± 30.1 SD (Table 3).

### Major attributes – regression results

Bivariate logistic regression analysis was used to identify candidate variables for multiple logistic regression and examined the unadjusted association between consistent delivery of ENC and the independent variables. The results obtained from this analysis are reported in Table 4. Availability of essential obstetric drugs in delivery rooms, CSC performances, availability of maternity waiting homes, consistent partograph use, BEmONC signal functions, availability of newborn corners, having separate delivery rooms, providing women-friendly delivery services, availability of required laboratory investigations for ANC, and establishing case review/audit system for maternal and newborn deaths were found to have significant association with CD-ENC with *p* < 0.05. In addition to the variables that came to significance on bivariate analysis, the independent variable—availability of trained provider—was taken as significant from the program implementation perspective with *p* < 0.257 (Crude Odds Ratio (COR) = 1.260, 95%CI: 0.845, 1.879).

**Table 4:** Factors associated with CD-ENC, multiple logistic regression analysis

	B	SE	Wald	df	AOR	95% CI	P-Value
Availability of trained providers (1)	0.12	0.23	0.25	1	1.12	0.71—1.77	0.62
Consistent partograph use (1)	0.98	0.22	18.95	1	2.66	1.71—4.13	0.00^a^
Availability of required laboratory investigations for ANC (1)	0.19	0.24	0.61	1	1.21	0.75—1.93	0.44
Availability of maternity waiting homes (1)	0.57	0.26	4.70	1	1.78	1.06—2.98	0.03^a^
Administration of BEmONC signal functions (1)	0.29	0.27	1.11	1	1.33	0.78—2.28	0.29
Separate delivery rooms (1)	0.62	0.42	2.15	1	1.86	0.81—4.24	0.14
Availability of essential obstetric drugs in delivery rooms (1)	0.63	0.25	6.42	1	1.88	1.15—3.08	0.01^a^
Availability of newborn corners (1)	0.10	0.26	0.14	1	1.10	0.66—1.84	0.71
Established case review/audit system for maternal and newborn deaths (1)	0.17	0.23	0.54	1	1.19	0.75—1.88	0.46
CSC performances	-0.01	0.00	9.17	1	0.99	0.98—1.00	0.00^a^
Provision of women-friendly delivery services (1)	0.81	0.34	5.71	1	2.24	1.16—4.35	0.02^a^
Constant	-2.05	0.58	12.52	1	0.13		0.00

^a^Statistically significant

*ANC* Antenatal care, *BEmONC* Basic Emergency Obstetrics and Neonatal Care, *CD-ENC* Consistent Delivery of Essential Newborn Care, *CSC* Community Score Card, *SD* Standard Deviation

In the multicollinearity check, none of the independent variables exhibited collinearity. The VIF value of all the 19 predictors were below 5 and there were no variables having an eigenvalue of > 0.90. Linearity of the continuous variables, distance between facility and woreda capital (KM) and community scorecard, with respect to the logit of the dependent variable were assessed via the Box-Tidwell (1962) procedure. A Bonferroni correction was applied using all the 19 terms in the model resulting in statistical significance being accepted when *p* < 0.00263 [19]. Based on this assessment, the continuous independent variables were found to be linearly related to the logit of the dependent variable.

A binomial logistic regression was performed to assess the effect of several factors on the likelihood that facilities practiced consistent delivery of ENC. The model contained 11 independent variables, (availability of trained providers, consistent partograph use, availability of required laboratory investigations for ANC, availability of maternity waiting homes, separate delivery rooms, BEmONC signal functions, availability of essential obstetric drugs in delivery rooms, availability of newborn corners, established case review/audit system for maternal and newborn deaths, CSC performances, and women-friendly delivery services). The logistic regression model was statistically significant, X^2^ (11) = 71.188, *p* < 0.0005. The model explained 21.2% (nagelkerke R^2^) of the variance in CD-ENC status, and correctly classified 72.0% of cases. Sensitivity was 87.5% and specificity was 44.1%. Of all cases predicted as applying CD-ENC, 73.8% were correctly predicted and the negative predictive value was 66.3%.

As shown in Table 4, five of the independent variables made a statistically significant contribution to the model, (availability of essential obstetric drugs in delivery rooms, CSC performances, availability of maternity waiting homes, consistent partograph use, and women-friendly delivery services). The strongest predictor of CD-ENC was consistent partograph use, recording an odds ratio of 2.66 (Adjusted Odds Ratio (AOR) = 2.66, 95%CI: 1.71, 4.13). This indicated that facilities that had consistent partograph use were 2.66 times more likely to practice consistent delivery of ENC, controlling for all other factors in the model. Similarly, providing women-friendly services was strongly associated with increased likelihood of exhibiting CD-ENC. Facilities with essential obstetric drugs also had 1.88 (AOR = 1.88, 95%CI: 1.15, 3.08) times higher odds of exhibiting consistent delivery of ENC.

## Discussion

The overall practice of facilities consistently applying ENC found in this study to be (64%) is comparable to the observational study reported in the northern part of Ethiopia where the overall practice of health care providers was 59.8% [5]. This study found five factors—availability of essential obstetrics drugs in delivery rooms, high community score card (CSC) performances, availability of maternity waiting homes, consistent partograph use, and availability of women-friendly delivery services—were important to ensure consistent delivery of essential newborn care at health center level.

Availability of trained providers, unlike in this study, was reported as a key factor for observed changes in practicing ENC [5, 8, 20]. In contrast, as reported by a study conducted in Bihar, India, training alone may not make staff capable of using various equipment required for ENC. The study reported that 20% of medical officers and 15% of nurses were not able to use Ambu bags, radiant warmers, oxygen concentrators, and suction machines despite their training [10]. Despite this, there are some studies that show that the assignment of trained providers is important. A study conducted in Ethiopia underlined that a significant proportion of facilities included in the study, (74%) had trained providers, but only 44% of those trained were assigned to the delivery rooms [21]. Given these contradictions, the role of training, and the distribution and assignment of trained providers in various types of facilities may require further studies. In addition to trainings and placement of staff, findings from this study indicate that interventions which create opportunities for close interactions with patients and the community are important predictors for the consistent delivery of ENC [22].

The availability of maternity waiting homes which allows mothers to stay in and around the facility is an opportunity for enhancing interactions between health workers and mothers [23, 24]. As reported by various studies, women reported MWHs as very important institutions to creating opportunities for and facilitating communication platforms between providers and mothers to elevate trust, build mothers' confidence, and promote bonding [16, 25, 26]. Additionally, they help health workers to adjust their schedule and ensure the availability of the required supplies and drugs [27].

Many studies report client-provider relationship and communication as relevant elements of maternal satisfaction and service utilization [28]. Health workers providing delivery care play a crucial role in establishing a link between the natural and technical dimensions of birth. Any care provided to mothers requires prompt responses to issues which may arise [16]. As reported by various studies, consistent partograph use is a sign of staff commitment to regularly monitor progress and take action for positive birth outcomes [29]. In line with these findings, consistent use of partograph during delivery care was found to be a key factor of signaling a consistent delivery of ENC by health workers. In addition to the health workers’ commitment, a labor monitoring tool which also has an integrated format for ENC, may serve as a reminder for health workers to accomplish all required tasks in relation to newborn care.

Readily available essential obstetric drugs and most importantly their proximity to the actual service delivery person in the room was found to be crucial. Having the required commodities for care in delivery rooms was a determinant factor for consistent delivery of ENC. In many of the cases—as demonstrated by other studies—essential medicines for newborn care, such as vitamin K [10, 21, 30], chlorhexidine [21, 31] and tetracycline eye ointment [21] are not available in similar settings. Availability of essential drugs for care is also dependent on various factors, such as supply chain logistics, provider attitude, and restrictions on use [32].

The working environment at health facilities [33] and negative attitudes of health workers towards pregnant women directly affects the quality of care [30]. Regularly monitoring these and striving for improvement is an influencing factor on the likelihood of success for any facility. Community scorecards strategize to create a platform to ensure the feedback of the community are presented to the health workers and health facility management through different mechanisms [34]. A facility with high scores translates as having relatively met the demands of the community in relation to availability of supplies, transportation facilities, waiting time and reception of health providers. As found in this study, the accountability platform which is being implemented in the form community scorecards is also another predictor of the provision ENC. A high score indicates that facilities are continuously applying improvements to address the community’s concerns, in terms of quality of care which is also a predictor for the consistent delivery of ENC.

Reading through the results from the study, it is good to note some of the limitations. The study used program monitoring data which was collected by a project staff. In addition, the methodologies used have some limitation as retrospective document review was used, and the results relied on the completeness of patient records as well as program monitoring database. In addition, the analysis was only able to control the effects of background information which is available with the authors.

## Conclusion

The delivery of essential newborn care depends on both health provider and facility manager actions and availability of platforms to streamline relationships between the clients and health facility management. Establishing maternity waiting homes within a facility strengthens the relationships between mothers and health workers and influences the completeness of care for each newborn. In addition, improving the friendliness of healthcare providers in delivery care and consistent use of various tools, such as partographs are important factors to note. The availability of essential drugs and their proximity to where the essential services are provided also contributes to the provision of ENC. Platforms like community score cards assess the level of client’ satisfaction and enable management teams to work towards improving the consistent delivery of care.

## Data Availability

The datasets during and/or analyzed during the current study are available from the corresponding author on reasonable request.

## Abbreviations

BEmONC: Basic Emergency Obstetrics and Newborn Care
CASH: Clean and safe health facility
CSC: Community Scorecard
CD-ENC: Consistent Delivery of Essential Newborn Care
DHIS: District Health Information System
ENC: Essential Newborn Care
FMOH: Federal Ministry of Health
HBsAg: Hepatitis B surface antigen
PHC: Primary Health Care
PHCU: Primary Health Care Unit
PITC: Provider Initiated Testing and Counseling
RPR: Rapid Plasma Regain
SD: Standard Deviation
USAID: United States Agency for International Development
VDRL: Venereal Disease Research Laboratory
VIF: Variance Inflation Factor
WHO: World Health Organization
